# Precision oncology through next generation sequencing in hepatocellular carcinoma

**DOI:** 10.1016/j.heliyon.2025.e42054

**Published:** 2025-01-17

**Authors:** Sayali Shinde, Carola Maria Bigogno, Ana Simmons, Nikita Kathuria, Aruni Ghose, Vedika Apte, Patricia Lapitan, Shania Makker, Aydin Caglayan, Stergios Boussios

**Affiliations:** a Barts Cancer Institute, Queen Mary University of London, Cancer Research UK Barts Centre, London, UK; b Department of Medical Oncology, St. Bartholomew's Hospital, Barts Health NHS Trust, London, UK; c British Oncology Network for Undergraduate Societies (BONUS), UK; d Faculty of Biology, Medicine and Health, University of Manchester, Manchester, UK; e QIAGEN Manchester, Manchester, UK; f Faculty of Life Sciences and Medicine, King's College London, London, UK; g Department of Medical Oncology, Medway NHS Foundation Trust, Kent, UK; h Department of Medical Oncology, Mount Vernon Cancer Centre, East and North Hertfordshire NHS Trust, London, UK; i University College London Medical School, London, UK; j University College London Oncology Society, London, UK; k School of Medical Sciences, The University of Manchester, Manchester, UK; l Division of Genetics and Epidemiology, The Institute of Cancer Research, Surrey, UK; m University College London Cancer Institute, London, UK; n Barts and the London School of Medicine and Dentistry, Queen Mary University of London, London, UK; o Barts and the London Oncology Society, London, UK; p Faculty of Life Sciences & Medicine, School of Cancer & Pharmaceutical Sciences, King's College London, Strand, London, UK; q Kent and Medway Medical School, University of Kent, Canterbury, UK; r Faculty of Medicine, Health, and Social Care, Canterbury Christ Church University, Canterbury, UK; s AELIA Organization, 9th Km Thessaloniki–Thermi, 57001 Thessaloniki, Greece

**Keywords:** Hepatocellular carcinoma, Next generation sequencing, Artificial intelligence, Precision oncology, Virus

## Abstract

Hepatocellular carcinoma (HCC) is a primary liver cancer that originates from underlying inflammation, often associated with Hepatitis B virus (HBV) or Hepatitis C virus (HCV) infections. Despite the availability of treatments, there are high rates of tumour relapse due to the development of drug resistance in infected cells. Next-Generation Sequencing (NGS) plays a crucial role in overcoming this issue by sequencing both viral and host genomes to identify mutations and genetic heterogeneity. The knowledge gained from sequencing is then utilised to develop countermeasures against these mutants through different combination therapies. Advances in NGS have led to sequencing with higher accuracy and throughput, thereby enabling personalized and effective treatments. The purpose of this article is to highlight how NGS has contributed to precision medicine in HCC and the possible integration of artificial intelligence (AI) to bolster the advancement.

## 1 Introduction

Hepatocellular carcinoma (HCC) accounts for around 80 % of liver cancers. It is the sixth most common cancer and the third leading cause of mortality from cancer illness worldwide [[1], [2], [3]]. Despite the overall incidence of HCC has declined since the start of the century, global disease burden is still significant (Supplementary Fig. 1) [4,5].

Hepatitis B virus (HBV) and Hepatitis C virus (HCV) account for the major risk factors in HCC, where HBV is responsible for about 50 % of the cases [6,7]. Amongst patients with active HBV or HCV infection, 2–5% are expected to develop HCC each year [8,9]. Alongside HBV and HCV, other factors leading to HCC consist of metabolic dysfunction-associated fatty liver disease (MAFLD), metabolic dysfunction-associated steatotic liver disease (MASLD) and progressive familial intrahepatic cholestasis (PFIC); few of which are discussed briefly in this review.

Management of HCC is multidisciplinary, guided by tumour staging and Child-Pugh score (Supplementary Table 1) [10,11].

Despite the many advances in diagnosis and management of HCC, several challenges span across different aspects of care, and include:

1. **Diagnosis** presents hurdles due to the limited sensitivity of imaging criteria, often necessitating invasive biopsies for conclusive results, further complicated by the difficulty in distinguishing high-grade dysplastic nodules from well-differentiated HCC [12].
2. **Surgical resection**, while effective in well-selected patients, faces limitations in those with underlying cirrhosis or clinically significant portal hypertension, impacting postoperative outcomes [13].
3. **Liver transplantation** criteria have evolved from restrictive morphologic criteria like the Milan criteria to include biological tumour behaviour considerations [14], although challenges persist in defining optimal alpha-fetoprotein (AFP) cut-off values and incorporating tumour grading [15].
4. **Systemic therapy** has seen notable advancements with the introduction of sorafenib and subsequent drugs, yet response assessment remains challenging, and predictive biomarkers for treatment efficacy are lacking.

While addressing such challenges, the emergence of new agents adds complexity to treatment decisions, requiring careful consideration of optimal sequencing and patient selection for improved outcomes in HCC management [13].

In this article, we highlight the development of next generation sequencing (NGS) as a precision medicine tool in the HCC landscape, a brief pathophysiological perspective, and the integration of artificial intelligence (AI) in further advancing this technology.

## 2 Deciphering disease: a pathophysiological perspective

A combination of chronic inflammation and genetic alterations is believed to contribute to the uncontrolled cell growth in the liver tissues, which ultimately results in HCC [16,17]. Viral hepatitis firstly leads to the immune system attacking infected hepatocytes, therefore causing local inflammation and leading to an increased cell turnover in an attempt to replace the infected hepatocytes. In chronic HBV and HCV, the liver tissue is constantly inflamed, and hepatocytes undergo oxidative stress. This, alongside increased rates of cellular apoptosis and turnover, leads to cirrhosis and fibrillogenesis. These structural changes alongside genetic alterations, predispose the liver tissue to further dysplasia, malignant changes and lastly HCC [[16], [17], [18], [19]].

### 2.1 pRb and cell cycle control

G1 to S phase transition is required for cells to undergo synthesis and cell division. Cyclins and cyclin-dependent kinases (Cdk) are responsible for these transitions. These kinases are required for the phosphorylation of retinoblastoma protein (pRb) in the late G1 phase. Phosphorylated pRb then activates the E2F family, leading to the upregulation of genes in the S-phase. pRb mutations are a common cause of most cancers [20,21]. In a South Korean study on 231 samples of frozen tissues from HCC patients, it was observed that cell cycle mutations for pRb and E2F were identified in 22 % of cases. Amongst them, pRb mutation was about 10 % due to HBV-related cases [22].

### 2.2 Replicative senescence and telomerase reverse transcriptase (TERT)

Replicative senescence is a way to reduce tumour suppression. However, mutations within the cells cause the cells to exit from this state of replicative senescence and re-enter the cell cycle which is related to the reactivation of telomerase [23]. In a study carried out between immortal cell lines, cancer cell lines, and normal cell lines, the presence of telomerase activity was more prominent in immortal and cancer cell lines than that in normal cell lines [24]. TERT enzyme responsible for the length of the telomerase is necessary for reactivation of telomerase activity. Mutations in *TERT* are one of the primary causes of HCC [25].

### 2.3 TP53 and apoptosis

The second most mutated gene in HCC patients is *TP53* and the common mutations are missense mutations, Eventually, leading to the loss of *TP53* function or the gain of functions such as angiogenesis which lead to tumour formation [26]. About 31 % of the tumours consisted of *TP53* mutations taking place constantly at two kinases, which are ATM and RPS6KA3 [25].

### 2.4 Virus host interactions

**Fig. 1:**
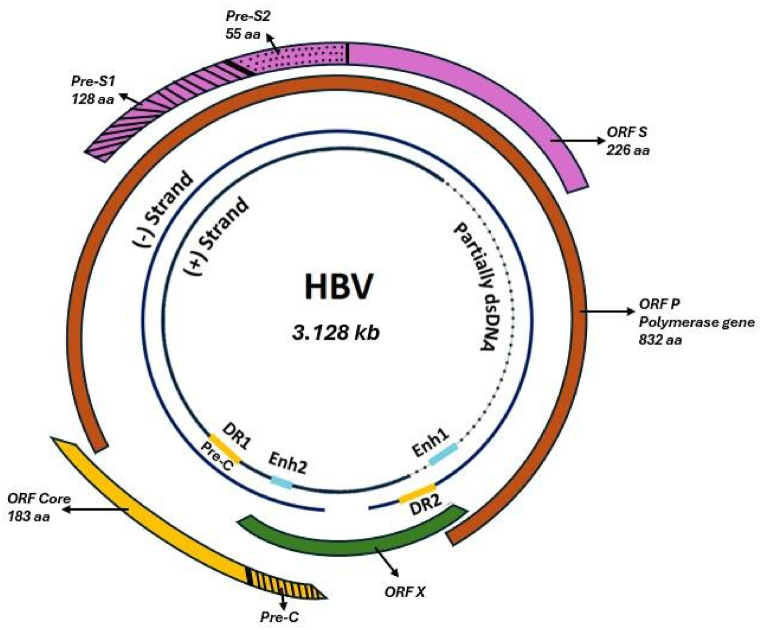
Structure of HBV genome [Adapted from 32].

HBV infects cells via encoding for a specific Hepatitis B surface antigen (HbsAg) (Fig. 1). HBV has a partly double-stranded relaxed circular DNA (rcDNA), which can integrate within the host DNA causing mutations and encodes for genes that protect HBV against the host immune response [27]. In a cloning study carried out in HCC patients during the early stage of development, it was observed that HBV integrates within the cyclin A gene. As cyclins are associated with the pRb gene it can lead to malignant cell growth [28]. HBV infection also increases the risk of HCC in the absence of prior liver damage from cirrhosis.

HBV is a DNA virus with a 3.2 kb genome. It is partly double-stranded **(**dotted line**)**. The gene S is responsible for coding the HBsAg protein. Sequences upstream from the S gene consist of pre-S domains (S1 and S2) which code for the virus receptor required for infection. Gene C codes for hepatitis B core antigen (HBcAg) which is responsible for the nucleocapsid of the virus. Gene P codes for the enzyme DNA-dependent DNA polymerase activity which is required for viral replication. X gene (HBx) is important since it is responsible for gene expression and protects the viral cells against the host's immune response.

**Fig. 2:**
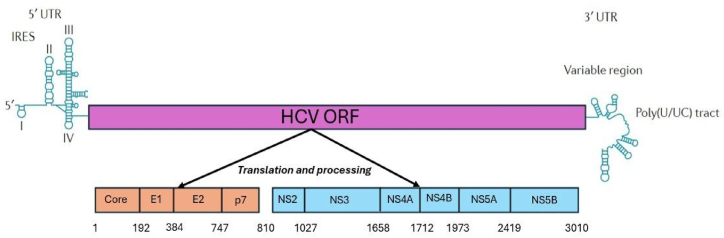
Structure of HCV genome [Adapted from 28].

In contrast, HCV is a single-stranded, positively charged RNA virus which does not integrate within the host DNA although the infection itself takes place due to underlying causes such as liver damage. The genetic material of HCV maintains itself as an endoplasmic-reticulum-associated episome. The virus then infects and reassembles itself in the hepatocytes and encodes for proteins via viral untranslated regions (UTR) that leads to cell survival and cell growth causing tumorigenesis (Fig. 2).

Both HBV and HCV are speculated to infect the cells when they are in the G0 state of the cell cycle. Usually, this leads to cell death, but when these viruses infect cells with defective negative growth regulatory circuitry, apoptosis does not occur, thus leading to the development of HCC [29].

HCV is a single-stranded RNA virus with a 9.6 kb genome that codes for 3000 amino acids from a single open reading frame (ORF). ORF consists of two untranslated regions (UTRs) viz. structural and non-structural regions. These are responsible for viral proteins and RNA synthesis. IRES is also known as the internal ribosomal entry site in the 5′UTR. From ORF, a variety of structural proteins are encoded, including core proteins, and two envelope glycoproteins (E1, E2) and p7. The latter is responsible for viral assembly once inside the host cell. Non-structural proteins NS2 and NS3 undergo cleavage and activate NS3 serine protease. NS3 is then responsible for the cleavage of NS4A, NS4B, NS5A, and NS5B. NS5B is responsible for the enzyme RNA-dependent RNA polymerase which replicates the viral RNA.

### 2.5 Additional drivers of HCC

Although HBV and HCV most commonly lead to HCC, other diseases such as PFIC and MASLD can also lead to liver cirrhosis and HCC. PFIC refers to a diverse group of autosomal recessive liver disorders that typically manifest in childhood, often presenting with hepatocellular cholestasis during the neonatal period. These conditions progressively lead to liver failure, with affected individuals usually facing severe complications and death from liver failure between infancy and adolescence. PFIC results from genetic defects in bile transport and secretion, causing intrahepatic cholestasis that can advance to end-stage liver disease. Genetically confirmed cases of PFIC account for 12%–13 % of cholestatic disorders in infants and children [30,31].

MASLD formerly classified as non-alcoholic fatty liver disease (NAFLD), is an increasingly prevalent global health issue, affecting over a quarter of the world's adult population. MASLD is closely linked to cardiometabolic risk factors such as obesity, type 2 diabetes, hypertension and is associated with higher rates of cardiovascular disease, chronic kidney disease, and various cancers. The disease spectrum ranges from simple steatosis (isolated fatty liver) to metabolic dysfunction-associated steatohepatitis (MASH), previously termed non-alcoholic steatohepatitis (NASH), an inflammatory form that can progress to liver fibrosis, cirrhosis, and hepatocellular carcinoma (HCC). Over the past decade, MASLD-related cirrhosis cases requiring liver transplants have increased tenfold. The pathogenesis of MASLD is complex, involving excess lipid accumulation, lipotoxicity, immune-mediated inflammation, and factors like genetic variants. Although not all patients with fatty liver develop severe outcomes, there is a lack of reliable biomarkers to predict progression, making personalized care challenging [32]. We will be discussing briefly about the developments made with the use of NGS in these diseases.

Understanding the pathophysiology of these diseases and viral infections is important as they cause genetic alterations that can lead to HCC. Identifying and analyzing these genetic changes is essential for effective diagnosis and treatment. This is where NGS becomes invaluable. NGS enables comprehensive examination of genetic mutations, aiding in the identification of potential biomarkers and the development of targeted therapies for HCC. The following section explores the advancements in NGS and its critical role in improving HCC management.

## 3 Next generation sequencing

NGS is a high-throughput solution for genetic screening, offering crucial insights into mutations within the viral genome, transmission networks, and mechanisms of drug resistance. NGS builds upon the foundation of Sanger sequencing, with modern systems sequencing several terabases in a single run. Indeed, a significant advancement compared to the 800-base limit of the Sanger method. The massively parallel processing capability of NGS is vital when investigating multiple targets, providing accelerated workflows and comprehensive coverage of low-frequency genetic variants, with call validation rates reaching 99.97 % [33].

Genetic sequencing is categorised into different levels based on successive generations of technology. The first generation, Sanger sequencing, is now less frequently used due to limitations associated with poorer prognosis. The second generation includes widely used techniques such as Roche 454, Ion Torrent, and Illumina sequencing. And finally, the third-generation sequencing offers real-time sequencing and includes technologies like Pacific Biosciences (PacBio) and Nanopore-based sequencing from Oxford Nanopore Technologies (ONT), such as MinION. These third-generation technologies are extensively used in conjunction with Illumina sequencing [34].

Despite the low yield of newer methods, Sanger sequencing remains the *‘*gold standard’ for verifying genetic variants due to its high sensitivity. This process relies on chain-termination in a method known as sequencing by synthesis. It uses a combination of standard deoxynucleotide triphosphates (dNTPs) and fluorescently labelled dideoxynucleotides triphosphates (ddNTPs), with the latter terminating extension when incorporated, producing fragments of varying lengths with fluorescently marked termini.

**Fig. 3:**
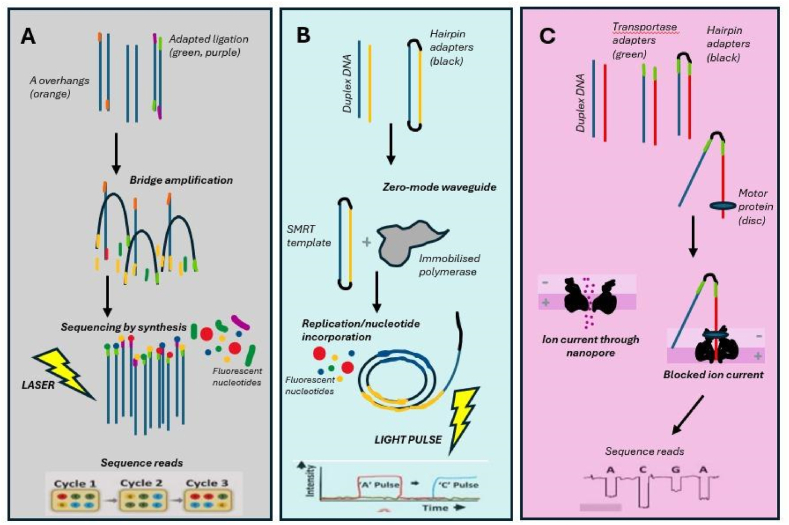
Sequencing technologies [Adapted from 37] (A) Illumina sequencing follows sequencing by synthesis method. This involves the denaturation of DNA, adaptor ligation, and bridge amplification which takes place on a flow cell surface. All 4 nucleotides are included which are individually labelled with different fluorophores which are useful for detection. (B) PacBio sequencing works on single-molecule real-time (SMRT) technology. The DNA molecule forms a loop with the help of hairpin adapters and is sequenced in a zero-mode waveguide which allows the detection of individual fluorophores in real-time with continuous light-pulse-based detection. (C) Nanopore sequencing uses a protein nanopore that is fixated on an electrically resistant polymer membrane. Electric current is passed through the membrane. A change in the current is observed when a DNA/RNA is passed through the nanopore and this allows the identification of sequences in real time.

The most widely used technique, Illumina sequencing, shares the sequencing by synthesis principle with Sanger sequencing. However, Sanger sequencing uses capillary electrophoresis to distinguish individual bases based on the position of the incorporated terminator dye, while Illumina sequencing, rely on a flow cell platform to sequence several genomes in parallel. Illumina employs the bridge amplification method (Fig. 3A). The process of library preparation ensures genetic samples are 'readable' on the sequencer platform through a series of steps, including fragmentation. This is followed by the addition of adapters necessary for high-level multiplexing and adhesion to the flow cell. Rapid bridge amplification generates isolated library clusters that are sequenced through the addition and imaging detection of fluorescently tagged dNTPs. Building on Sanger technologies, Illumina sequencing cleaves the terminator elements of the incorporated bases, allowing multiple incorporation cycles to be captured in a single run [33,34].

Another second-generation platform, the Ion Torrent utilises a semiconductor to detect the release of protons during nucleotide addition. It's working principle resulted in higher speed and lower cost. Shortly after the advancement of the second-generation platforms, third generation sequencing emerged with the distinctive features such as single-molecule sequencing and sequencing in real-time, wherein we could sequence the nucleotide molecules without the need for PCR amplification of the template. The PacBio sequencing method, also known as single-molecule-real-time (SMRT) sequencing is the first ever nano sensor-based technology (Fig. 3B). Another approach developed by the ONT, called the nanopore sequencing detects the changes in the electric current caused due to the disorder of the nanopore particles when nucleotide strands pass through them. Thus nanopore sequencing is directly able to identify the changes in the electric current in real-time during the sequencing run (Fig. 3C) [35].

Additionally, liquid biopsy has emerged as a non-invasive technique that detects mutations with the help of NGS using circulating tumor DNA (ctDNA) from the blood samples. Liquid biopsy platforms like Guardant 360 CDx and FoundationOne Liquid CDx have been approved by the FDA for cancer diagnostics and treatment monitoring. Guardant 360 CDx and FoundationOne Liquid CDx are both qualitative in vitro diagnostic devices that use NGS to detect genetic alterations. These platforms analyse ctDNA obtained from a simple, non-invasive blood draw. Guardant 360 CDx identifies single nucleotide variants (SNVs), insertions, and deletions in 55 genes, while FoundationOne Liquid CDx detects and reports substitutions, insertions, and deletions across 311 genes [36,37]. These technological advancements enable the real-time monitoring of tumor dynamics, detection of minimal residual disease, and identification of resistance mutations, enhancing personalized treatment strategies for diseases such as HCC which are discussed further in this review. Technical specifications regarding the most frequently used sequencing platforms can be found in Table 1.

**Table 1:** Technical specifications regarding the different Next Generation Sequencing (NGS) sequencing platforms.

Platform	System	Use	Sequencing Technology	Throughput (per day)	Read length	Turnaround time	Reference
Illumina	HiSeq 2000 (dual flow cell)	Short read sequences	Sequencing by synthesis	55 Gb	2 × 100bps	∼11 days	[38,39]
Ion Torrent	Ion GeneStudio 5S	Short read sequences	Sequencing by synthesis	15 Gb	200bps	∼19 h	[38,40]
PacBio	Revio	Short read sequences	Sequencing by binding	90 Gb	20–25 kb	∼30 h	[38,41]
Nanopore DNA sequencing	MinION	Long read sequences	Sequence detection by electrical impedence	48 Gb	>4 Mb	∼72 h	[38,42]

NGS has greatly transformed the analysis of genetic mutations in HBV and HCV, offering detailed insights into HCC. NGS enables precise viral and host genotyping, which is crucial for identifying specific mutations and developing personalized treatments.

### 3.1 Viral genotyping and treatment implications

#### 3.1.1 HBV

The HBV has a covalently bonded closed circular double-stranded DNA. Frequent mutations occur due to the absence of the 3′-5′ proofreading function of DNA polymerase, leading to the generation of viral quasispecies responsible for drug resistance [43]. NGS focuses on detecting quasispecies in various regions, including the HBV reverse transcriptase (RT) region, pre-C/core region, pre-S/S region, or full-length sequencing [44].

An acute infection in the pre-core C (pre-C) region, specifically the *G1896A* mutation, can lead to hepatic failure. Illumina Genome Analyzer II has been employed to identify mutations in *G1896A* associated with HBe serostatus.

Antiviral drugs like lamivudine (LMV), adefovir (ADV), entecavir (ETV), tenofovir (TDF), and telbivudine are used for HCC treatment. These nucleoside analogue drugs block the HBV DNA polymerase, which inhibits the viral replication. Lamivudine is an oral 2′,3′-dideoxynucleoside which inhibits the DNA synthesis by terminating the nascent proviral DNA chain. The concentrations used for Lamivudine are specific towards blocking the HBV DNA and limits any off-target effects. ADV is an oral pro-drug for Adefovir which is a nucleotide analogue of adenosine monophosphate. ADV inhibits reverse transcriptase and DNA polymerase activity and gets incorporated into the HBV DNA leading to chain termination. ETV is an analogue of 2′-deoxyguanosine. It can inhibit HBV replication by, inhibiting the priming of HBV DNA polymerase and also inhibition of reverse transcription of negative strand HBV DNA and synthesis of the positive strand HBV DNA. TDF is an oral prodrug of tenofovir. TDF is an nucleoside analogue with a potent activity against HBV DNA polymerase. Telbivudine is a thymidine nucleoside analogue which is phosphorylated intracellularly to active triphosphate form. This activated form competes with the natural substrate thymidine 5′-triphosphate to inhibit HBV DNA polymerase and thus reducing viral DNA replication [45]. These nucleoside analogues are widely used however long-term use can result in antiviral drug-resistant HBV. Sequencing has revealed that ETV can reduce *G1896A* mutations, while *M204VI* and *M250VI* mutations occur during LMV and ETV therapy. ADV-resistant mutations at *A1*81TV and *N236T* have also been detected. Sequencing these mutations helps identify reasons for resistance against nucleoside analogue therapy [43]. Mutations in the core promoter region, such as *A1762T* and *G1764A*, reduce Hepatitis B e antigen (HBeAg) levels and are associated with continuous replication of HBV DNA, which may lead to HCC [44]. Due to Illumina sequencing limitations for short lengths, Nanopore technologies are employed for genome-length reads [46].

#### 3.1.2 Managing antiviral resistance mutations in HBV

NGS leads to the detection of minor variants which are not easily picked up by traditional sequencing methods. In a study, Betz-Stablein et al. [47], performed single-nucleotide sequencing using NGS platforms to investigate HBV variants in a patient suffering from chronic hepatitis B infection, which would eventually lead to HCC. The patient had a liver transplant followed by different treatment regimens using antiviral drugs. Samples were taken from the liver explant and blood over a period of 15 years. NGS helped them to identified multiple mutations in the HBV polymerase gene (e.g., *rtL180M, rtT184S, rtM204V*), which are associated with resistance to LMV and ETV [47].

The study also highlights how clinicians can keep a track of evolution of viral quasispecies overtime and prevent future drug resistance with the help of NGS. Due to LMV/ETV resistance mutations, the patient was switched to ADV monotherapy which helped in maintaining these mutations. However, with the help of NGS, they were able to identify how the viral population rapidly accumulates nonsynonymous mutations under antiviral pressure. New mutations (*rtM204A* and *rtN2*36 A/V) had emerged, which were previously undescribed in LMV or ADV resistance. This led to reduced sensitivity towards LMV and could also contributed to ADV resistance [47]. By identifying these mutations early through NGS, physicians can adjust treatment regimens before the resistant viral strain becomes dominant [48].

This allows clinicians to select more effective drugs, such as switching to TDF monotherapy when resistance to LMV or ETV is detected. Studies have shown that TDF monotherapy is just as effective as TDF-based combination therapy in maintaining viral suppression in LMV-resistant patients [49]. For example, in a study of 90 patients with ETV resistance, both TDF monotherapy and TDF-ETV combination therapy successfully suppressed HBV replication [50].

In cases of ADV resistance, ETV monotherapy has also proven to be effective. A randomized study of 102 patients comparing TDF monotherapy with TDF-ETV combination therapy found that both approaches were equally effective in managing ADV-resistant chronic hepatitis B [50]. As a result, ETV monotherapy or TDF-ETV combination therapy can be considered for ADV resistance [49].

However, managing multi-drug resistance can be more challenging. In a European multicenter study, TDF monotherapy was effective in achieving a sustained antiviral response in patients with resistance to both LMV and ADV [51]. Additionally, the combination of ETV and TDF has been shown to be highly effective in rescuing patients with treatment failure after exposure to multiple antiviral drugs. Therefore, with the help of NGS, understanding the specific mutation profile of an individual patient is possible, which can help clinicians tailor a personalized antiviral therapy [52].

#### 3.1.3 HCV

HCV is an RNA virus with core proteins, envelope proteins E1 and E2, p7, and nonstructural proteins NS2, NS3, NS4A, NS4B, NS5A, and NS5B. Lack of proofreading activity in RNA-dependent RNA polymerase during replication leads to nucleotide alterations and genetic variability.

Antivrial therapy regimes consisting of Peginterferon (PegIFN-*α*) and Ribavirin (RBV) were commonly used in combination (known as PR therapy) for the treatment of HCV infection. PegIFN-*α* is a modified form of interferon-alpha. It leads to the activation of immune cells such as natural killer cells and macrophages and enhances their ability to target and destroy HCV DNA. RBV has an direct antiviral effect against the HCV RNA-dependent RNA polymerase. It acts as an inhibitor of inosine mono-phosphate dehydrogenase which leads to the depletion of guanosine triphosphate pools. RBV also leads to lethal mutagenesis of the viral load leading to the ineffective production of virus [53].

DAA therapy has been proven to be more prominent than the PR therapy. The main mechanism of action of DAAs is the inhibition of enzymes such as protease or polymerase but there are other DAAs which inhibit the assembly of the replication complex, for example, NS5B inhibitors and NS5A inhibitor. Telaprevir and Boceprevir are the commonly used DAAs which inhibit the viral NS3/4A serine protease which is important for replication. However, drug resistance is still considered a problem while treating HCV infections which can be identified by NGS [54]. NGS is used to determine viral quasispecies and drug-resistant mutants insensitive to direct-acting antiviral (DAA) or interferon-based treatments. Ultra-deep pyrosequencing (UDPS) identifies mutations in the interferon sensitivity-determining region in NS5A associated with drug resistance [55]. Amino acid substitutions in viral protein regions like NS5B polymerase or NS3/4A protease inhibitors lead to drug resistance against STAT-C. This has necessitated combination treatments involving STAT-C, interferon, and RBV [56].

#### 3.1.4 Managing antiviral resistance mutations in HCV

Previously Ji et al. has used NGS particularly through tagged pooled pyrosequencing which led to the detection of drug resistance mutations in HCV such as S282G, S282R, and S282C, which were not detected by conventional methods. They found out that the spatial and charge-related amino acid changes observed in these variants affects the binding to a NS5B inhibitor; sofosbuvir leading to drug resistance. Molecular modelling further supported the role of these variants in potential resistance mechanisms, providing a valuable tool for predicting drug-resistant polymorphisms [57].

In another study by Kim et al. data from 36 Korean patients who had failed first-line DAA therapy were analyzed using NGS to guide adjustments in treatment regimens. The study found that NS5A mutations, such as Y93H and L31, were linked to DAA failure in patients with genotype 1b infection. Failure was primarily observed with two-drug combinations like daclatasvir and asunaprevir (DCV + ASN). After identifying these resistance-associated substitutions (RASs) through NGS, clinicians switched to a more potent triple combination therapy consisting of NS5B inhibitor, NS5A inhibitor and NS3 inhibitor; sofosbuvir, velpatasvir, and voxilaprevir (SOF/VEL/VOX), which was highly effective and achieved a 100 % sustained virological response (SVR) [58]. This regimen is specifically designed to overcome NS5A and NS3 RASs [59].

For patients who failed NS5A inhibitors like DCV + ASN, NGS detection of NS5A mutations also informed clinicians to avoid using regimens such as glecaprevir/pibrentasvir (GLE/PIB) in certain cases. This decision was based on findings that NS5A RASs did not significantly impact GLE/PIB's efficacy in genotype 1b patients. However, for those with prior NS5A inhibitor failure, SOF/VEL/VOX remained the preferred retreatment option [58].

Additionally, the study observed NS5B S282T RAS in genotype 2 patients who had failed treatment with sofosbuvir and ribavirin (SOF + RBV). In these cases, treatment with GLE/PIB was recommended for those who experienced sofosbuvir failure. NGS also helped correct genotyping errors in two patients, ensuring they received the most suitable therapy for their correct genotype [58]. By ensuring more tailored therapies, NGS contributed to higher SVR rates, even in patients who had previously experienced DAA failure.

### 3.2 Genotyping in PFIC and MASLD

NGS has been efficient in determining mutations in diseases such as PFIC and MASLD. Previously, the three most prominent conditions in PFIC were known which were PFIC 1,2 and 3. Mutations in genes like *ATP8B1* (causing PFIC 1), *ABCB11* (PFIC 2), and *ABCB4* (PFIC 3) were identified as key drivers of PFIC. However, about two-thirds of patients with normal gamma-glutamyl transpeptidase (GGT) cholestasis, typically seen in PFIC, do not carry mutations in these genes. This led to the discovery of three new conditions; PFIC 4, 5, and 6, caused by mutations in *TJP2* (tight junction protein 2), *NR1H4* (farnesoid X receptor), and *MYO5B* (linked to microvillous inclusion disease), respectively [30].

Clinically, NGS has significant implications as it enables early and accurate diagnosis of these rare cholestatic conditions, which are crucial for targeted treatment and management. For instance, identifying *NR1H4* mutations can help predict early-onset vitamin K unresponsive coagulopathy, while *TJP2* mutations are linked to a higher risk of HCC. NGS has become the primary choice for testing PFIC phenotype due to it being non-invasive in comparison to liver biopsy and IHC. It is also suggested to further test the samples for whole exome (WES) or whole-genome (WGS) sequencing for proper interpretation due to the complexity of the disease [30].

For the detailed analysis of MASLD, Kendall et al. designed the first comprehensive data platform; SteatoSITE specifically for MASLD research. It integrates a wide range of data, including RNA sequencing (RNA-seq) of liver tissues, quantitative histopathological assessments, and clinical data from electronic health records (EHRs). The sequencing data was obtained with the help of Illumina NovaSeq 6000 platform [32].

Through NGS-based RNA-seq, the researchers used SteatoSITE to identify key mutations and gene expression changes associated with MASLD progression. They uncovered important gene networks, or regulons, linked to fibrosis and disease outcomes. Mutations in genes such as *CHRDL2, STC1, CTGF, GDNF*, and *FGF7*; which are involved in liver fibrosis and inflammation were highlighted, shedding light on their potential role in advancing MASLD towards more severe stages, including liver cirrhosis and hepatocellular carcinoma (HCC). The dataset also helped develop a risk-prediction model based on hepatic gene expression, improving the prediction of liver decompensation, a key turning point in MASLD progression. By providing this integrated resource, SteatoSITE advances precision medicine in MASLD by enabling the identification of biomarkers and therapeutic targets specific to different disease stages [32].

### 3.3 Host genotyping and treatment implications

Whole-genome sequencing (WGS) of HCC patients reveals mutations in several host genes, including *TERT*, *TP53*, *CTNNB1* (beta-catenin), *AXIN1*, *ARID1A*, and *WWP1*. *TERT* exhibits the highest mutation rate at approximately 60 %, followed by *TP53* with mutations ranging from 35 to 50 %, and *CTNNB1* with mutations ranging from 19 to 40 % [60].

TKIs such as sorafenib are commonly used in HCC. However, due to mutations, activation of the PI3K-mTOR pathway results in resistance to these agents. Sorafenib-resistant HCCs utilize the PI3K-mTOR pathway as a bypass route, necessitating the inhibition of this pathway to restore sensitivity [61]. Additional TKIs, such as lenvatinib, can be used in combination with other TKIs [62]. The combination of atezolizumab (anti-programmed death ligand 1, anti-PD-L1) monoclonal antibody and bevacizumab (anti-vascular endothelial growth factor, anti-VEGF) monoclonal antibody has proven to be more effective than sorafenib [62]. Mutations in beta-catenin lead to oncogenic WNT signalling and resistance to immune-based therapies, such as anti-PD-L1 and anti-cytotoxic T lymphocyte-associated protein-4 (anti-CTLA-4) monoclonal antibody [61]. Mutations were also detected in host chromatin regulatory genes, including *ARID1A*, *ARID1B*, *ARID2*, *MLL*, *MLL3*, *BAZ2B*, *BRD8*, *BPTF*, *BRE*, and *HIST1H4B*. WGS determined that the loss of function in these genes also contributes to the progression toward HCC [63]. Recent advancements in molecular research have unveiled promising biomarkers for predicting prognosis and guiding therapeutic decisions in HCC. Here, we summarize key findings from notable studies, highlighting molecular biomarkers and their prognostic implications in HCC. Table 2 presents a comprehensive overview of these recent molecular discoveries and their impact on prognostic assessment and therapeutic strategies for HCC patients [64].

**Table 2:** Recent molecular findings and prognostic impact in Hepatocellular Carcinoma (HCC).

Reference	Biomarker	Sample size (patients/tissue samples with HCC)	Significant Findings
[65]	*TERT*	305	*TERT* genetic alterations can be used as a biomarker for early diagnosis of HCC
[66]	*TP53*	865	● Development of a prognostic model sensitive to *TP53* mutations for HCC patients ● The model can differentiate between low to high risk of unfavourable survival
[61]	*WNT/β-catenin* pathway and *TP53*	127	● NGS in HCC patients showed *PI3K–mTOR* pathway alterations in sorafenib-treated patients and *WNT/β-catenin* signaling alterations in immune checkpoint inhibitor-treated patients associated with poorer outcomes ● Potential guidance for treatment
[67]	*CTNNB1*	Data obtained from cbioportal	● Mutated *CTNNB1* can be used as a biomarker for HCC patients undergoing immunotherapy ● It may distinguish between responders and non-responders
[68]	*CCNB2*	148	*CCNB2* identified as prognostic factor in HCC development, promoting cell proliferation through *CCNB2/PLK1* pathway
[69]	*KIF* family	295	Significant associations with tumour progression and prognosis, suggesting *KIFs* as potential prognostic and therapeutic biomarkers for HCC
[70]	*CDK1*	320	● *CDK1* was identified as an independent prognostic factor and potential therapeutic target for HCC ● In cellular models, inhibition of *CDK1* showed promising effects on reducing cellular proliferation and viability in HCC cells

### 3.4 Clinical implications of NGS in HCC detection

#### 3.4.1 Limitations of traditional HCC detection methods

The optimal imaging modality for detecting HCC remains a topic of debate. Ultrasound (US) and AFP are widely used for screening due to their broad availability and low cost. However, the accuracy of US varies significantly, largely depending on the operator's experience, scanning technique, and equipment used. A major limitation is the poor sensitivity of US for detecting small nodules, particularly in early-stage HCC [71].

Current guidelines recommend abdominal US, with or without AFP testing, every six months for HCC surveillance. While US shows moderate sensitivity (58–89 %) and high specificity (90 %) for detecting liver cancer, it still misses up to 40 % of early-stage HCC cases [72]. While testing the serum AFP levels, they are quite lower in the setting of a solitary HCC lesion measuring less than 2 cm in size compared with larger lesions [73,74]. Increased AFP levels can also be seen in the absence of HCC, including chronic liver disease without HCC, non-hepatic malignancies and normal pregnancies. This can further lead to false-positive results [73].

Magnetic resonance imaging (MRI) is considered the gold standard for diagnosing HCC, particularly when a suspicious lesion is detected on US. MRI confirms malignancy based on criteria such as lesion size (≥10 mm) and specific blood flow patterns like arterial enhancement and venous washout. However, MRI has limitations, especially in identifying malignant lesions smaller than 10 mm. While the sensitivity of MRI for lesions larger than 20 mm has improved to 96 % with updated criteria, detecting smaller, early-stage tumours remains challenging. Additionally, small HCC lesions in cirrhotic livers often lack the typical blood flow patterns required for diagnosis. For lesions between 1 and 2 cm, the sensitivity drops to around 71 % [72]. Therefore, due to these technical difficulties, many precursors or early forms of HCC are missed through these criteria.

#### 3.4.2 The role of NGS in enhancing HCC diagnosis

These challenges focus on the need for additional parameters to improve risk assessment through the use of precision medicine. Precision medicine is where we use a multiomics approach and patients are no longer treated solely based on histology of their tumour but instead through actionable targets specific to their tumour biology. Performing NGS with the help of tissue samples was done quite traditionally. NGS panels like MSK-IMPACT enhance cancer care by providing publicly accessible tumour data for research and identifying actionable mutations for clinical decision-making [75].

For precision oncology in patients mainly with solid tumours, Memorial Sloan Kettering Institute developed the Memorial Sloan Kettering-Integrated Mutation Profiling of Actionable Cancer Targets (MSK-IMPACT) [76]. It is a hybridization capture-based NGS assay for targeted deep sequencing of all exons and selected introns of 341 key cancer genes in formalin-fixed paraffin-embedded tumours (FFPE). This assay is authorized by the New York State Department of Health and the FDA as a clinical test. This test was successfully used by Harding et al. and they found the potential biomarkers which can help in clinical diagnosis of HCC patients [61]. They used tissue samples from patients confirmed with histological diagnosis of advanced and metastatic HCC. Samples were sequenced with targeted NGS using MSK-IMPACT by Illumina Hiseq 2500 platform. They found mutations which activate the PI3K-mTOR pathway are associated with poor outcomes in sorafenib-treated patients. Sorafenib-resistant HCCs utilize PI3K-mTOR as a potential bypass pathway, and PI3K inhibition via RNA interference or small-molecule inhibitors restores sorafenib responsiveness. In addition, mutations which activate the WNT pathway are associated with innate resistance to immune checkpoint blockade. Mainly tumours harbouring activating *CTNNB1* and inactivating *AXIN1* mutations are related to disease progression. These potential biomarkers along with others mentioned in Table 2 can help in clinical diagnosis of HCC [61].

While NGS via tissue samples is efficient for molecular profiling, it is an invasive method and typically not used for early-stage HCC screening. This highlights the importance of liquid biopsy, a non-invasive technique using blood samples for sequencing, offering a less intrusive alternative for early detection and monitoring. A recently developed approach of liquid biopsy instead of the traditional tissue sampling for sequencing methods can prove to be useful in early-stage detection of HCC [72]. Liquid biopsy focuses on identifying new targets by utilizing cell-free DNA (cfDNA) or ctDNA.

Although detectable at a low concentration in healthy individuals, cfDNA is found in significantly higher concentrations in patients with chronic inflammatory or malignant diseases. Detectable cfDNA in a considerable amount has been reported in HCC patients, and in patients with liver cancer. The potential for the analysis of cfDNA variants carried by cirrhotic patients may indicate abnormal changes towards a malignant development at a stage when HCC is not yet recognizable by standard imaging [72].

In a recent study by Alunni-Fabbroni et al., they used cfDNA obtained from blood samples from patients involved in the advanced HCC phase II SORAMIC trial [77]. NGS was performed using Illumina 150 paired-end mode. The commonly detected mutations were observed in *TP53* and *CTNNBI*. Other driver mutations consist of *BAX* and *HNF1A*. *BAX* belongs to the Bcl-2 gene family of pro-apoptotic proteins and inactivation of *BAX* might be a mechanism of escape death. *HNF1A* codes for a transcription factor (*HNF1α*) which is necessary for liver development and differentiation. It functions as a tumour suppressor and its inactivation can play an important role in tumour development. In addition, patients with post-operative high levels of cfDNA had a shorter survival. This was mainly observed several weeks after sorafenib treatment, suggesting that in advanced HCC cfDNA can help in monitoring therapy response [77].

In another study led by Alunni-Fabbroni et al., they found of the progression of HCC from liver cirrhosis samples using cfDNA. They performed mutation profiling of cfDNA isolated from liver cirrhosis patients without HCC according to US and AFP. NGS was performed with the help of Illumina platform. At the time of blood analysis, patients were diagnosed with liver cirrhosis without any suspicion of hepatic malignancy. However, in 22 % of patients carrying genetic variants, re-evaluation of imaging revealed suspicious liver lesions, with four patients progressing to HCC. Importantly, mutations were detected before the precursor lesions developed into HCC [72].

In line with findings in HCC, frameshift mutations were more frequently observed in genes like *HNF1A* (28 %), *BAX* (25 %), and *ASXL1* (19 %), with identical codon changes seen in HCC. *ASXL1*, a scaffold protein, has been implicated in various cancers, such as breast, hematologic, and prostate cancers, also acting as a tumor suppressor. A follow-up MRI study one year later revealed new HCC lesions in two patients, which had been missed in the initial scans. These findings, in dysplastic nodules, emphasize the value of cfDNA for early HCC detection [72].

Combining NGS analysis of cfDNA with traditional methods such as MRI could enhance the detection of early-stage HCC, particularly for high-grade dysplastic nodules and lesions smaller than 10 mm, which may not exhibit clear malignant features on imaging alone. In comparison to traditional HCC screening methods such as US, AFP, and MRI, NGS platforms like Illumina, though initially more expensive, offer significant long-term advantages. Technical aspects for the commonly used platforms like Illumina and others can be found in Table 1. While US and AFP are more cost-effective, they often lack the sensitivity required for early-stage detection, and MRI, though more accurate, comes with higher operational costs. NGS, particularly via non-invasive liquid biopsy, enhances early detection by identifying actionable mutations, potentially reducing unnecessary treatments. Although the upfront cost of NGS is higher, it may result in overall cost savings by enabling early diagnosis and guiding personalized treatment strategies, which can reduce expenses associated with late-stage disease management.

While there are various methods for detecting HCC, it is essential to follow established guidelines to prevent overdiagnosis. Unlike false positives, overdiagnosis refers to the detection of true positives that are not clinically significant findings that, if left untreated, would not have caused harm to the patient. To mitigate overdiagnosis in terms of HCC, it is necessary to focus on using strict diagnostic criteria such as LI-RADS (Liver Imaging Reporting and Data) system and also guideline recommendations put forth by the American Association for the Study of Liver Diseases (AASLD) and European Association for the Study of the Liver (EASL) for semi-annual surveillance for at-risk patients [78].

While NGS can identify numerous mutations in HCC, including those for which no effective treatments exist, this does not necessarily mean it leads to overdiagnosis. Instead, it can be viewed as over detection or identification of mutations which currently do not have any clinical significance. However, the clinical utility of detecting these mutations depends on the context. For example, identifying the prominent biomarkers such as *TP53*, *TERT* and *CTNNB1* can help refine treatment strategies, guiding doctors to avoid ineffective therapies. Even if certain mutations do not have immediate therapeutic options, they can still provide valuable prognostic or surveillance data, helping to monitor disease progression and inform future treatment decisions as new therapies are developed.

### 3.5 Integrated liquid biopsy and tissue sampling

The advent of NGS technologies has undeniably marked a milestone in personalized medicine and drug discovery [79]. However, further studies assessing its direct impact and benefits, particularly in the management of HCC, are still required.

NGS was first introduced in 2005 and has significantly enhanced our understanding of cancers, particularly those linked to viral causes such as HCC and HBV/HCV [80]. It has enabled scientists and clinicians to deepen their knowledge of genomic sequences, quasispecies, and medication-resistant mutations, leading to more specific and effective management plans tailored to the patient, their cancer, and their viral infection [81].

We now comprehend HCC pathophysiology and disease progression better, thanks to valuable data from NGS. However, there are also several limitations.

Firstly, HCC exhibits a low prevalence of actionable target mutations compared to other cancers. NGS has identified *TERT*, *CTNNB1*, *TP53*, *AXIN1*, *ARID1A*, and *ARID1B* as the most prevalent mutations, although these cannot be easily targeted by novel therapies. Furthermore, NGS has mostly focused on clinical targets identified in formalin-fixed paraffin-embedded tumour tissue. A further challenge in HCC is the high variability of tumour heterogeneity, which can present as (1) intrapatient, among patients with a similar tumour history, (2) intertumor, related to multiple tumour foci in the same patient, (3) intratumor, when differences are found within the same tumour cluster, and (4) treatment-associated heterogeneity, describing changes arising at different times throughout treatment [81].

However, it is believed that focusing on 'liquid biopsy,' including cfDNA or circulating tumour cells, might be a more effective way of identifying new targets. Moreover, integrating liquid biopsy alongside traditional tissue sampling methods can provide more comprehensive understanding of HCC. Liquid biopsy which includes sampling cell-free DNA or circulating tumour cells from the blood, offers a non-invasive and potentially more accessible method for detecting tumour-specific mutations and heterogeneity. It could be a convenient method for primary care doctors to monitor for liver cancer therefore aiding in HCC surveillance. Liquid biopsy has a potential to eventually replace or work alongside imaging tests for monitoring HCC patients. It can also help in diagnosis of liver nodules that are difficult to identify with imaging tests. Liquid biopsy could also help doctors to see how well treatments are working at any stage of liver cancer and help them adjust treatment plans as needed [79]. The development of liquid biopsy, exemplified by sequencing tools like Guardant360 has revolutionized HCC diagnosis offering a non-invasive method for detecting tumour specific mutations. By analysing cfDNA, Guardant360 can detect key mutations associated with HCC, such as *TERT, CTNNB1, TP53* and others [82]. In summary, the integration of liquid biopsy and tissue sampling in HCC diagnosis (Table 3) and monitoring represents a significant leap forward, enhancing the effectiveness of treatment across all stages of the disease.

**Table 3:** Comparison between tissue sampling and liquid BIOPSY.

*ASPECT*	Tissue Sample	Liquid Biopsy
*SAMPLE TYPE*	Liver tissue obtained via needle biopsy	Blood sample collected via standard venipuncture
*PROCEDURE*	Invasive procedure, carries risks of bleeding, infection, and other complications such as needle tract seeding	Minimally invasive, relatively safe
*SAMPLE COLLECTION*	Requires skilled personnel for tissue extraction and preservation	Standardized blood collection, suitable for routine procedures
*SAMPLE VOLUME*	Provides larger sample size for comprehensive analysis	Provides small volume of circulating tumour components
*SAMPLE QUANTITY*	Adequate for extensive molecular analysis	Limited, may require multiple samples for comprehensive analysis
*SAMPLE QUALITY*	High quality, preserves tissue architecture and integrity	Subject to degradation, may affect molecular analysis
*TUMOUR HETEROGENEITY*	May capture tumour heterogeneity but subject to sampling bias	Captures tumour and tumour microenvironment heterogeneity
*SENSITIVITY*	High sensitivity to detect molecular alterations	Sensitivity affected by tumour size, may miss early-stage lesions
*SPECIFICITY*	Specific to tissue type, provides accurate diagnosis and access to spatial information	Variable specificity, affected by background noise
*TURNAROUND TIME*	Longer processing time due to tissue processing and analysis	Faster turnaround time, suitable for rapid diagnosis
*COST*	Higher cost due to invasive procedure and extensive analysis	Lower cost, especially for routine monitoring
*PLATFORMS FOR NGS PROFILING*	Illumina, Ion Torrent, PacBio	Guardant360 CDx, FoundationOne Liquid CDx

## 4 Integrating NGS and AI

Artificial Intelligence was first introduced in the 1950s and has since evolved rapidly, especially during the 21st century. Researchers and clinicians have been able to apply it to all fields of healthcare in this todays time [83].

**Fig. 4:**
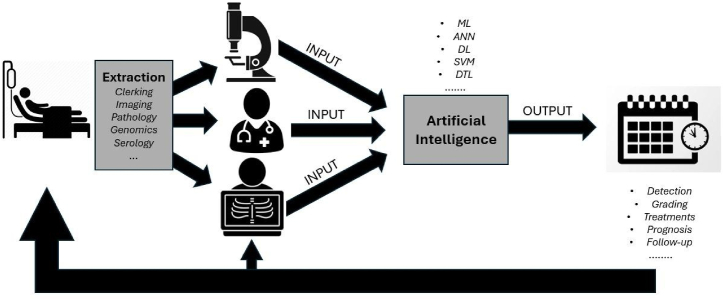
Schematic representation of how AI has been utilised by researchers and clinicians in detection and management of diseases such as HCC [Adapted from 87].

AI has been utilised in cancer research including HCC to assess, combine and interpret the “big data” around imaging, serology and histopathology for early detection, prognosis and personalized management plans for HCC treatment. This process is achieved through the data, information, knowledge, wisdom (DIKW) framework which gathers data and then analyses and compares it to other existing DIKW frameworks [84]. For instance, studies have shown that early detection from AI algorithms correlates to better prognosis because of earlier and targeted intervention (Fig. 4) [85]. Furthermore, HCC is typically diagnosed from liver biopsies, however AI can analyse radiology scans and diagnose the tumour early from specific radiological features with no need for further biopsies without delaying the treatment. Thus, leading to improving patient outcomes [86].

Therefore, it would seem natural to apply AI to the interpretation of NGS results as well. The foundational layer of data serves as the fundamental level, with information adding depth, knowledge introducing the concept of how to apply it, and wisdom determining the optimal timing and circumstances for its utilisation [87]. This framework extends to areas such as machine learning (ML), deep learning (DL), neural networks, and big data. More specifically, deep learning methods and neural networks alike can be utilised to analyse large databases of data efficiently and precisely, to interpret complex genomic profiles as well as to identify driver mutations, genomic alterations and potential drug targets commonly involved in the development of HCC such as *TP53*, to better inform and guide treatment [88,89]. AI-based algorithms are becoming increasingly available, including DeepVariant and Mutect2 amongst others [90].

In terms of real-life applications, studies and trials alike are showing that AI is making a significant impact in interpreting and comparing large datasets, identifying mutations and analyse tumour models, all information which is contributing to the future of personalized medicine. In turn, this information can lead to better diagnostic precision, predicting treatment response for individual patients and their specific tumour profile, and thus guide clinicians to design more informed and personalized management plans, which would and is already leading to better patient outcomes and decreased side effects and complications from aggressive cancer treatment [88].

The framework is instrumental in assessing the merits and drawbacks by comparing various researchers' models and conducting multidimensional analyses within specific medical fields related to HCC. For example, research has indicated that early intervention based on early HCC diagnosis can significantly enhance prognosis [82]. Furthermore, specific radiological features enable the diagnosis of HCC without the need for a confirmatory biopsy [86].

Despite the several benefits and undeniable potential of utilizing AI to interpret NGS data for HCC, there are also limitations and challenges which should be addressed. Firstly, data heterogeneity poses a significant challenge, since HCC is a highly heterogeneous disease showing significant variations between patients in terms of specific cancer mutations, tumours microenvironment and epigenetic changes [88]. Despite AI algorithms being trained to account for such variability, results may present biases. Secondly, said HCC complexity and variability often requires multi-omic data, including genomics, transcriptomics, epigenomics and proteomics [89,91]. Similarly to the challenges around data heterogeneity, despite AI algorithms being trained in integrating multi-omic data types, biases and noise may still be noticed the more variety is introduced and asked to integrate. Moreover, to fully validate the clinical application of AI in HCC, more large-scale, multicentre trials would be beneficial to assess the diverse AI models and their benefit and effectiveness in real-life patients [91].

## 5 Conclusion and future directions

HBV and HCV infections pose a significant risk for the development of HCC through chronic inflammation and oxidative stress, impacting a large population worldwide and contributing to substantial mortality. While antiviral medications offer treatment options, the high viral variability often leads to drug resistance. Consequently, medications initially highly effective can quickly become inefficient in managing the infection.

As also discussed by Schulze K et al. in their paper published in 2016, the primary goal of NGS is to sequence the viral quasispecies and determine genetic heterogeneity, which is crucial for developing sensitive drug therapies that resist resistance against the quasispecies, and they also highlight the advances and ongoing emerging potential of NGS in HCC diagnosis and treatment [92]. WGS faces a challenge in maintaining accuracy during sequencing between short and long-length reads. For instance, Illumina sequencing reads short lengths of the viral genome with high accuracy and throughput. On the other hand, Nanopore technologies sequence long genome lengths covering all mutations within the virus, albeit with low-fidelity data. ONTs like MinION MkII and PromethION are being developed to offer faster short-length reads with higher accuracy and coverage, aided by efficient algorithms for computational tools [93].

Similarly, the integration of AI in the domain of HCC is a rapidly growing phenomenon. Particularly in the field of cancer, AI plays a pivotal role in expediting precise diagnoses and managing vast datasets obtained from repositories such as the National Institute for Health and Care Excellence (NICE), as well as extensive genetic information from various HCC-related genes available on platforms like PubMed. AI serves as a key tool in enhancing early medical detection and crafting comprehensive treatment strategies for patients. As discussed by Schulze K et al., we agree that the molecular heterogeneity of HCC continues to pose a major challenge in the application of NGS to clinical practice, however AI is also certainly helping to breach the gap, and it will certainly continue to do so as more studies become available and the technology itself progresses.

## CRediT authorship contribution statement

**Sayali Shinde:** Writing – original draft. **Carola Maria Bigogno:** Writing – original draft. **Ana Simmons:** Writing – original draft. **Nikita Kathuria:** Writing – review & editing. **Aruni Ghose:** Writing – review & editing, Conceptualization. **Vedika Apte:** Writing – review & editing, Conceptualization. **Patricia Lapitan:** Writing – review & editing. **Shania Makker:** Writing – review & editing. **Aydin Caglayan:** Writing – review & editing. **Stergios Boussios:** Writing – review & editing, Supervision.

## Data availability statement

Data sharing is not applicable. No data was used for the research described in the article.

## Declaration of Competing Interest

The authors declare that they have no known competing financial interests or personal relationships that could have appeared to influence the work reported in this paper.
