# Supplementary material for: Precision oncology through next generation sequencing in hepatocellular carcinoma
Source: Heliyon. 2025 Jan 17;11(3):e42054. doi: 10.1016/j.heliyon.2025.e42054 (PMC11804570; doi:10.1016/j.heliyon.2025.e42054)
Supplement: Multimedia component 1 [file mmc1.docx]

**SUPPLEMENTARY MATERIALS**

**Supplementary Figure 1.** Incidence, 5-year prevalence and mortality rates of Hepatocellular Carcinoma (HCC) in 2020 worldwide, as provided by the Global Cancer Observatory, World Health Organization [3].

Figures A, B and C provide exceptional insight into areas worldwide affected by higher incidence rates, prevalence proportion and mortality rates, which could in turn be further amplified with migratory flows.


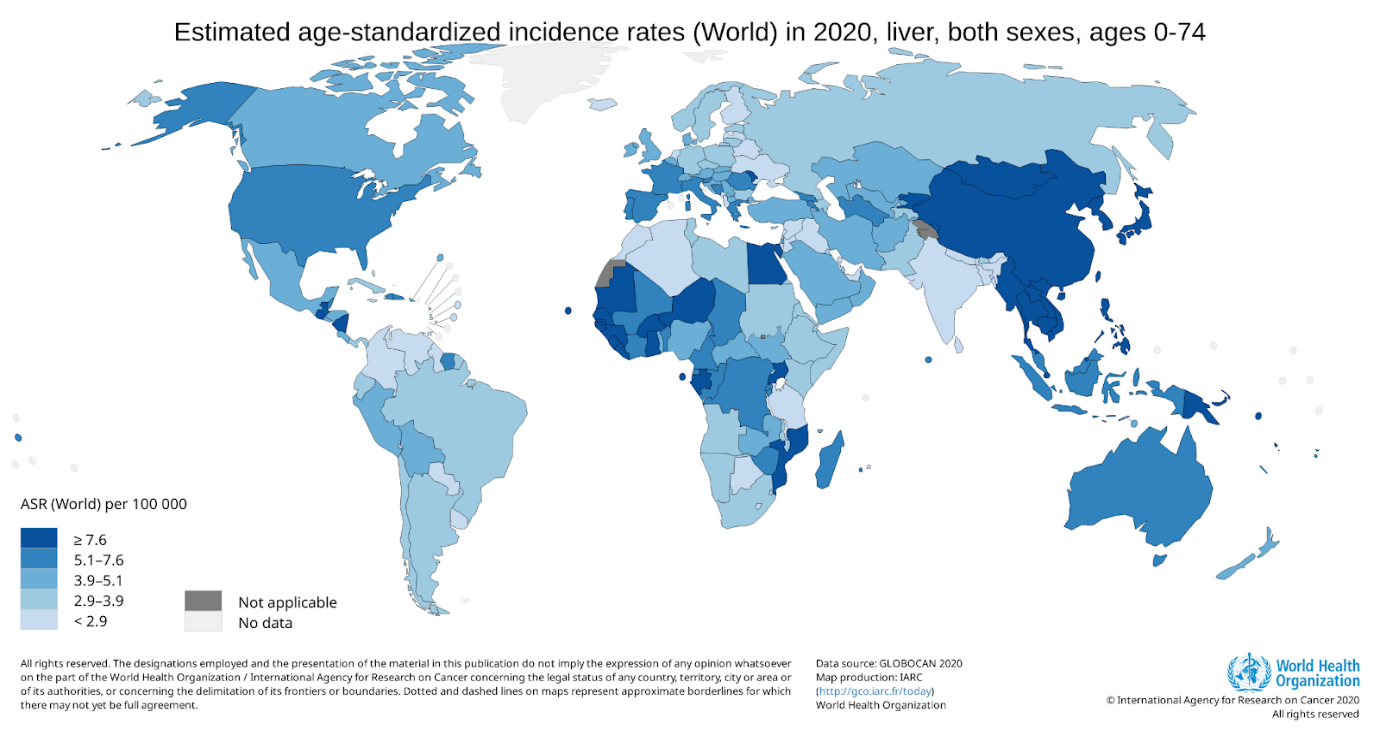


**Supplementary Figure 1 (A)** represents the estimated age-standardised (ASR, age group 0-74) incidence rates of HCC worldwide in 2020. It includes both sexes and identifies areas of the world with higher incidence (ASR ≥ 7.6 per 100 000 people) compared to lowest incidence (ASR ≤ 2.9 per 100 000 individuals). It also identifies areas where data is not applicable or not available.


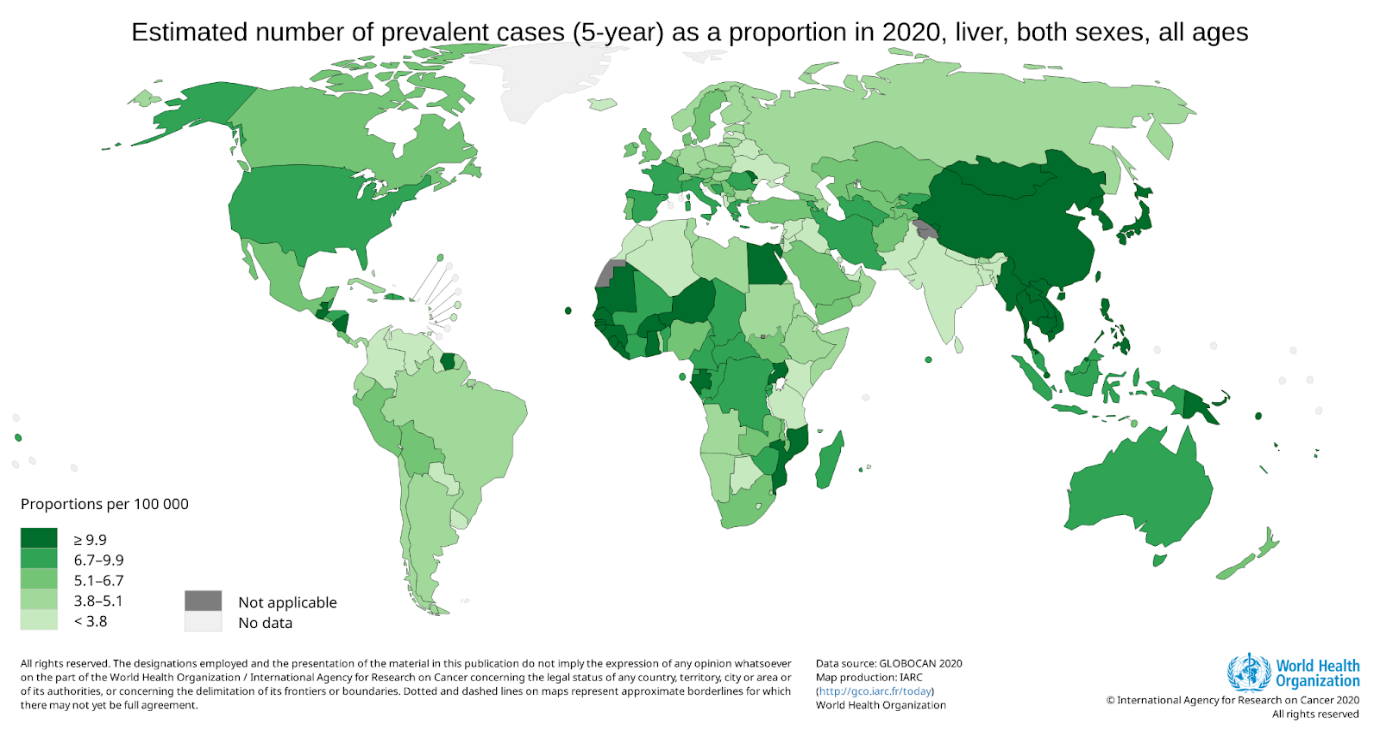


**Supplementary Figure 1 (B)** represents, as a proportion, the estimated 5-year prevalence of HCC worldwide in 2020, including both sexes and all ages. It also identifies areas of the world with higher prevalence (proportion ≥ 9.9 per 100 000 people) compared to lowest prevalence (proportion ≤ 3.8 per 100 000 individuals), as well as areas where data is not applicable or not available.


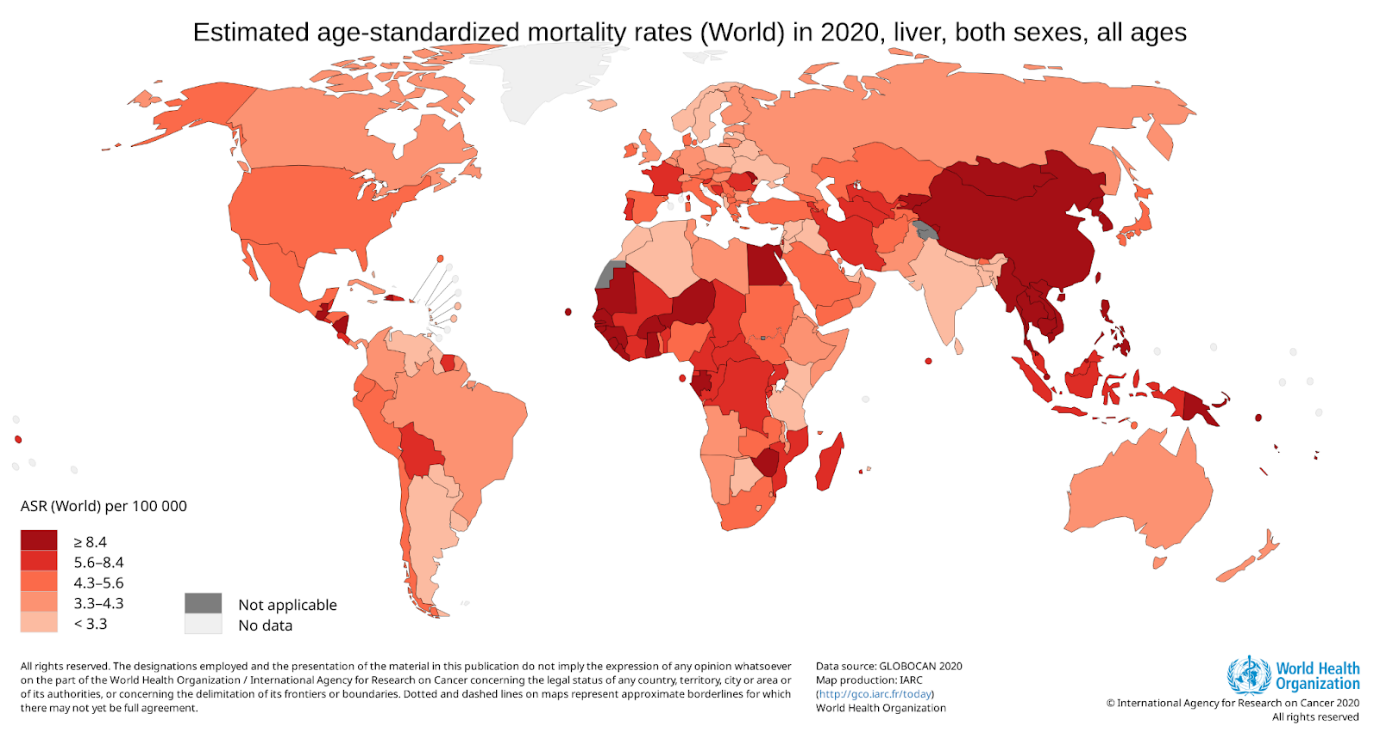


**Supplementary Figure 1 (C)** highlights the ASR mortality rates from HCC worldwide in 2020, including both sexes and all age groups. It identifies areas of highest mortality rates (ASR ≥ 8.4 per 100 000 people) compared to lowest incidence (ASR ≤ 3.3 per 100 000 individuals), as well as areas where data is not applicable or not available.

**Supplementary Table 1.** Treatment options based on staging of HCC

| Stage of HCC | Treatment | Reference |
| --- | --- | --- |
| Very early stage | - Radiofrequency ablation therapy | [12–14] |
| Early stage HCC | - Surgical resection - Liver transplant - Radiofrequency radiation therapy |  |
| Intermediate stage | - Ethiodized oil-based conventional trans-arterial chemoembolization (TACE) - TACE with drug-eluting beads (DEB-TACE) - Radioembolization - Selective internal radiation therapy (SIRT) |  |
| Advanced stage HCC | - Radioembolisation - Targeted systemic treatment (sorafenib) |  |
| End stage HCC | - Best supportive care - Palliative care - Palliative chemo/radiotherapy |  |
| Abbreviation: HCC - Hepatocellular Carcinoma | | |
